# Relationship between human tumour angiogenic profile and combretastatin-induced vascular shutdown: an exploratory study

**DOI:** 10.1038/sj.bjc.6604426

**Published:** 2008-07-08

**Authors:** A Gaya, F Daley, N J Taylor, G Tozer, U Qureshi, A Padhani, R B Pedley, R Begent, D Wellsted, J J Stirling, G Rustin

**Affiliations:** 1 Department of Clinical Oncology, Guy's & St Thomas' Hospitals, London SE1 7EH, UK; 2 Gray Cancer Institute, Northwood, Middlesex HA6 2RN, UK; 3 Paul Strickland Scanner Centre, Northwood, Middlesex HA6 2RN, UK; 4 Department of Medical Oncology, Royal Free Hospital, London NW3 2QG, UK; 5 Department of Statistics, University of Hertfordshire, College Lane, Hatfield, AL10 9AB, UK; 6 Department of Medical Oncology, Mount Vernon Hospital, Northwood, Middlesex HA6 2RN, UK

**Keywords:** combretastatin, immunohistochemistry, vascular maturity, dynamic MRI, microvessel density, angiogenesis

## Abstract

Combretastatin-A4-phosphate (CA4P) acts most effectively against immature tumour vasculature. We investigated whether histological angiogenic profile can explain the differential sensitivity of human tumours to CA4P, by correlating the kinetic changes demonstrated by dynamic MRI (DCE-MRI) in response to CA4P, with tumour immunohistochemical angiogenic markers. Tissue was received from 24 patients (mean age 59, range 32–73, 18 women, 6 men). An angiogenic profile was performed using standard immunohistochemical techniques. Dynamic MRI data were obtained for the same patients before and 4 h after CA4P. Three patients showed a statistically significant fall in *K*^trans^ following CA4P, and one a statistically significant fall in IAUGC_60_. No statistically significant correlations were seen between the continuous or categorical variables and the DCE-MRI kinetic parameters other than between ang-2 and *K*^trans^ (*P*=0.044). In conclusion, we found no strong relationships between changes in DCE-MRI kinetic variables following CA4P and the immunohistochemical angiogenic profile.

Combretastatin-A4-phosphate (CA4P) is a tubulin-binding, selective vascular disruptive agent (VDA) that causes rapid shutdown of blood flow to tumours with minimal effects on normal vasculature. Tumour endothelial cells are often immunohistochemically immature, lacking smooth muscle and pericyte coverage, and more sensitive to the effects of VDAs probably because the intracellular cytoskeleton is less developed (Tozer *et al*, 2005). Our aim was to determine human tumour vascular maturity using an immunohistochemical angiogenic profile, and to study any relationship with CA4P response assessed by change in DCE-MRI kinetic variables.

We selected antibodies to differentiate immature vascular endothelium – CD105 (endoglin), CD61 (*β*_3_-integrin), a pericyte marker α-smooth muscle actin (*α*SMA), a marker of hypoxia (Glut-1), proliferation marker (Ki-67), and markers associated with the angiogenic switch (VEGF, PDGFB, angiopoietin-1, and angiopoietin-2).

## Materials and methods

Following ethics approval, patients enrolled in Mount Vernon phase I CA4P studies (CR-UK PH1/066 & PH1/092) who underwent DCE-MRI analysis were identified. Records were obtained from 26 identified patients and tissue was available for 24 patients (Table 1). A full set of DCE-MRI data was available for 21. Tissue blocks were coded to conceal patient identity. Sections (4 *μ*m) were cut onto slides, and stained with the panel of antibodies at concentrations and with control samples illustrated in Table 2.

### Immunohistochemical procedures

All sections were deparaffinised in xylene and rehydrated through graded alcohols to water.

#### CD34 and *α*SMA double staining

Dako's Cytomation Envision double stain system (K5361) (Dako Ltd., Cambridgeshire, UK), was used performed according to the manufacturer's instructions. Specimens were counterstained using Gills haematoxylin (01500E; Surgipath Europe Ltd.) for 7 s and washed. The samples were mounted and cover slipped using Dako Faramount aqueous mounting medium (S3025).

#### CD105

We used Dako's Catalysed Signal Amplification staining system (K1497) and followed the manufacturer's instructions exactly.

#### VEGF, Ki-67, and Glut-1

Dako's Universal autostainer (Dako) was used. Antigen retrieval was performed in 0.01 M citric acid pH 6. Glut-1 and Ki-67 were microwaved for 3 × 4 min and VEGF for 4 × 4 min. Optimum antigen retrieval times were determined from titration experiments.

### Ang-1, Ang-2, PDGF, and CD61

Benchside manual staining procedures were used. Endogenous peroxidase activity was quenched with 1.5% hydrogen peroxide in methanol for 10 min. Antigen retrieval was performed by microwaving in 0.01 M citric acid pH 6 for 10 min (ang-1) and 20 min (ang-2). After washing in Tris-buffered saline tween-20 (TBST) primary antibody was applied as shown in Table 2, and incubated overnight at 4°C. After rinsing in TBST, Dako Envision anti-rabbit secondary antibody (K5007) with peroxidase was applied for 30 min. Antigen was developed with diaminobenzidine, slides counterstained with haematoxylin, dehydrated, cleared, and mounted. For CD61 and PDGF Vectastain's mouse IgG normal horse serum (Vector Labs, Burlingame, CA, USA) diluted in TBS was applied for 30 min. A horse anti-mouse biotinylated secondary was then applied, followed by incubation with streptavidin-peroxidase reagent.

### Immunohistochemical profile analysis

Each slide was assessed using a Zeiss Axioskop microscope, with Visilog 5.0 (Noesis) image capture software. The random field techniques described by Eberhard *et al* (2000) were used. Any CD34^+^ endothelial cells that were distinctly separate from other microvessels at high power were considered individual vessels, and recorded as the number of vessels per high power field. *α*SMA staining was analysed concurrently – any CD34^+^ vessel also *α*SMA^+^ was recorded. Mean microvessel density (MVD per mm^2^) and pericyte coverage index (PCI) were calculated after analysing at least 20 random fields per slide (Table 3). To determine the Ki-67 proliferation index (%) or Glut-1 hypoxia index (%) each slide was scanned at low and high magnification. A semiquantitative visual assessment was made across the slide of the proportion of tumour cells staining positively (Table 3).

The following antigens were analysed using a categorical scale, with the proportion of immunoreactive cells graded as weak, moderate, or strong by direct visual assessment at low ( × 50) and high ( × 400) magnification (Table 3). The most appropriate cutoff values were selected from previous studies; VEGF: absent (<5%), weak (5–25%), moderate (25–50%), and strong (>50%) (Erdem *et al*. 2007); Ang-1/2: cytoplasmic expression predominantly and grading system as for VEGF (Lind *et al*, 2005); PDGF: highly expressed in stroma; absent (<1% tumour staining), weak (1–10%), moderate (10–25%), and strong (>25%) (Barnhill *et al*, 1996).

Random results were checked by an experienced immunohistochemist (FD), who had not seen the initial data. In case of disagreement as to the correct result, that slide was analysed by a consultant histopathologist.

### DCE-MRI data

DCE-MRI involves the rapid acquisition of sets of T1-weighted images through tumour as an intravenous bolus of contrast agent is injected. The change in signal intensity over time can be analysed with mathematical models (Tofts, 1997). Quantitative kinetic variables are derived that provide information about tumour microcirculation as they are indirectly related to perfusion, vascular permeability, and vessel surface area (d'Arcy *et al*, 2006).

The IAUGC (initial area under the gadolinium concentration–time curve – units: mM min) is calculated for the first 60 s following Gd-DTPA injection (Evelhoch, 1999; Evelhoch *et al*, 2004). It has the advantage of being a quantitative variable that is obtained without mathematical modelling or knowledge of the arterial input function. It is a measure of how much contrast agent is taken up by the tumour in the first 60 s post-Gd-DTPA injection and is influenced by tumour blood flow rate and tumour vessel permeability. By using AUC from the Gd-DTPA concentration time curve rather than from the signal intensity time curve, the problem of dependence of signal enhancement on tissue T1 levels is avoided.

The transfer constants for Gd-DTPA diffusion into the tumour extravascular extracellular space (EES) from blood plasma (*K*^trans^) and back again (*k*_ep_) can be calculated using Tofts' mathematical model (Tofts and Kermode, 1991; Tofts *et al*, 1999). *K*^trans^ (and *k*_ep_) have both blood flow rate and permeability components as Gd-DTPA is not freely diffusible and their biological meaning is dependent on the balance between capillary permeability and blood flow in the tissue of interest. In high permeability tissues, *K*^trans^ is equal to the blood plasma flow per unit volume of tissue. In low permeability tissues such as the brain, it is equal to the permeability surface area product between the blood plasma and the EES per unit volume of tissue (Tofts, 1997). In most tumours the situation is likely to be somewhere between these two extremes.

Studies were performed on a 1.5 T scanner as previously described (Galbraith *et al*, 2003). Images were analysed using specialist software (Magnetic Resonance Imaging Workbench (MRIW), Institute of Cancer Research, London) (d'Arcy *et al*, 2006). A dynamic analysis was performed using MRIW for each pixel within the region of interest on each slice. The MRIW converts signal intensities of the T1-weighted DCE-MRI data set into T1 relaxation rates and then into Gd-DTPA concentrations for individual voxels, using the methods described by Parker *et al* (1997). Standardised terms were employed as defined in Tofts *et al* (1999). The percentage change from mean baseline kinetic variables following CA4P was calculated (Table 4).

### Statistical analysis

Measurement error (Bland and Altman, 1996) was used to define confidence intervals for the DCE-MRI variables. Linear regression and correlation analysis were used to test for relationships between the continuous variables and MRI data. Analysis of Variance was used to test for relationships between categorical variables and MRI data.

## Results

Tissue was received from 24 patients (mean age 59, range 32–73, 18 women, 6 men). Eighteen patients were taken from the PH1/066 single agent UK CA4P trial (Rustin *et al*, 2003), and 6 from the PH1/092 CA4P+anti-CEA antibody study (Gaya AM *et al*, 2008, unpublished). The most common histological subtypes (Table 1) were colorectal adenocarcinoma (7), uterine leiomyosarcoma (4), and ovarian adenocarcinoma (3). The age of the tumour blocks varied between 6 months and 21 years. All but one block contained primary tumour from surgical specimens, not metastatic tissue as patients were not rebiopsied before entrance to the phase I trials. We only studied patients who received doses of CA4P between 40 and 114 mg m^−2^ that have been associated with changes in DCE-MRI kinetic variables.

There was a wide range of DCE-MRI variable change following CA4P (Table 4). Percentage change in *K*^trans^ from mean baseline following CA4P varied between +111 and −75%, and IAUGC_60_ between +186 and −90%. In reality, only falls in these parameters (consistent with tumour vascular shutdown) are clinically significant. The 95% confidence interval for *K*^trans^ change (determined from repeatability analysis in the individual studies and repeated with the combined data) was −62.5 to +166.5%, IAUGC_60_±80%. There was no obvious visual relationship seen between CA4P dose and fall in kinetic parameters (Table 4). Three patients (3, 10, 12) showed significant falls in *K*^trans^ following CA4P (outside 95% CI), and only 1 patient (12) a significant fall in IAUGC_60_.

No statistically significant correlations were seen between the continuous or categorical variables and the DCE-MRI kinetic variables (Tables 5a and 5b) other than between ang-2 and *K*^trans^ (*P*=0.044). Inspecting Tables 3 and 4, there are no obvious relationships between the angiogenic profile and DCE-MRI data.

## Discussion

We found no obvious relationships between changes in DCE-MRI variables and the immunohistochemical angiogenic profile. This suggests immunohistochemical analysis of patients' tumours is unlikely to be useful in predicting response to VDA therapy. There was enormous variability seen both in the DCE-MRI kinetic variables and the immunohistochemistry. It is difficult to know whether the variability is related to the MRI and immunohistochemical techniques or is due to specific tumour characteristics that are of interest to us ie vascular maturity.

The variability of CD34 and CD105 MVD is consistent with previous studies. Mean microvessel density has been correlated with risk of metastasis, prediction of efficacy of anticancer therapy, and prognosis in a variety of tumour types (Des Guetz *et al*, 2006; Gadducci *et al*, 2006; Zhao *et al*, 2006; Barresi *et al*, 2007; Calvin *et al*, 2007; Gulubova and Vlaykova, 2007; Kavantzas *et al*, 2007; Trivella *et al*, 2007). The PCI results agree with Eberhard's data (Eberhard *et al*, 2000). We have found no evidence that mature vasculature with higher PCI responds poorly to CA4P, or that immature CD105^+^ vasculature responds better. Tumours with higher CD34 MVD may also have higher CD105^+^ MVD, however it does not necessarily follow that active angiogenesis takes place.

There are a number of aspects of this study that undermine the power of our conclusions. We obtained only small numbers of samples with a heterogeneous mix of different tumour types. However, our sample was limited to patients who had received CA4P and undergone DCE-MRI. Within the ANOVA categorical analysis especially, this greatly reduced the statistical power. Our paraffin blocks, up to 21-year old, were taken from the primary surgical procedure, whereas patients received CA4P during phase I trials many years later after metastatic relapse. It is still unclear whether antigenicity within paraffin blocks reduces over time (Fergenbaum *et al*, 2004). There are also suggestions that the angiogenic phenotype might continuously evolve from primary tumour through to metastasis (Sullivan and Graham, 2007).

There were widespread falls in MRI kinetic variables following CA4P, however for most patients these did not achieve statistical significance. For some this was because the CA4P dose received was below the threshold level for DCE-MRI detectable drug activity of 50 mg m^−2^ (Galbraith *et al*, 2003) – for others the CA4P simply did not have the expected tumour blood flow effect. The reason for administration of the lower CA4P doses in a few patients was the dose escalation protocol for the phase I trial that revealed this phenomenon. 95% CI for *K*^trans^ change was −62.5 to +166.5%, IAUGC_60_±80%. Conversely, taking the two separate trials individually, in PH1/066 the lower 95% confidence limit for *K*^trans^ was −44% and in PH1/092 it was −76.5%. This wide DCE-MRI parameter variability is in line with previously published data (Padhani *et al*, 2002; Taylor *et al*, 2004, 2006). Combining DCE-MRI data can adversely affect statistical confidence intervals due to the slight differences in sequences and calibration methods used (Taylor *et al*, 2006). There was wide intrapatient variability, patient motion, and an estimated arterial input function. Modified Fritz-Hansen arterial input coefficients, which are currently used, demonstrate better reproducibility (Fritz-Hansen *et al*, 1996; Taylor *et al*, 2007). Averaging the kinetic parameters over a whole lesion also dilutes the effect of CA4P central vascular shutdown because of relative sparing at the tumour periphery.

There was no obvious visual relationship between CA4P dose and fall in MRI parameters. Most patients treated with >50 mg m^−2^ show reductions in MRI variables, and examining those patients' results in Table 4 there is clearly no correlation, confirmed statistically. Several patients received lower, possibly inactive doses of CA4P. A threshold relationship for DCE-MRI detectable CA4P activity, with patients receiving >50 mg m^−2^ exhibiting greater parameter falls has previously been demonstrated (Galbraith *et al*, 2003; Rustin *et al*, 2003).

Microvessel density does not reflect the functional properties of vessels, including permeability, which contribute to DCE-MRI variables. Studies reporting correlation between MRI variables and MVD have therefore found only moderate associations (Su *et al*, 2003; Tuncbilek *et al*, 2004). Several studies (Buckley *et al*, 1997; Stomper *et al*, 1997; Su *et al*, 2003) show conflicting data relating immunohistochemical expression with DCE-MRI data. The different analysis methods used may have affected the results. Hawighorst *et al* (1998) showed that MRI parameters were better indicators of patient survival than MVD or VEGF.

The significant correlation between ang-2 and *K*^trans^ is probably a statistical quirk due to the number of variables investigated. However, it might be explained as: angiopoietins are involved in the angiogenic switch (Ahmad *et al*, 2001; Tanaka *et al*, 2003). Ang-1 stabilises vessels by maintaining pericyte coverage; ang-2 promotes pericyte removal, which in the presence of VEGF facilitates the angiogenic response and in the absence of VEGF induces vessel regression (Moon *et al*, 2006; Bach *et al*, 2007; Shim *et al*, 2007; Winter *et al*, 2007). Depending on the tumour model, stabilisation of blood vessels by ang-1 may either promote tumour angiogenesis or reduce tumour growth (Metheny-Barlow and Li, 2003). Wang (Wang *et al*, 2007) has shown ang-2 correlates with active angiogenesis in human colorectal carcinomas, and that ang-2 also correlates with VEGF. Thus, unstable immature ang-2 expressing vasculature undergoing neoangiogenesis is probably more susceptible to the effects of VDAs and thus detectable by DCE-MRI.

In conclusion, there was wide intrapatient variability in angiogenic profile expression and DCE-MRI kinetic variable changes following CA4P. There was no obvious correlation between falls in MRI variables and vascular markers, however sample numbers were small. Immunohistochemical analysis of patients' tumours is unlikely to be useful in predicting response to VDA therapy. This was an exploratory study, which we hoped would suggest some leads for further analysis. Although one could postulate that this study should be expanded to look at larger patient numbers and use fresh tumour samples from a homogeneous group, such a study is unlikely ever to be performed.

## Figures and Tables

**Table 1:** Patient demographics and histologies

**Patient ID**	**Patient age**	**Phase I trial**	**Tumour site**	**Histology**	**Date specimen taken**	**CA4P Dose (mg m^−2^)**
01	64	PH1/066 UK phase I single agent CA4P	Paratesticular	Spindle cell sarcoma	08/1999	52
02	51		Adrenal	Carcinoma	06/1993	68
03	65		Uterus	Leiomyosarcoma	03/1999	88
04	63		Renal	Renal papillary carcinoma	11/1997	40
05	49		Breast	Ductal carcinoma	02/1993	68
06	65		Uterus	Leiomyosarcoma	11/1982	52
07	71		Rectum		01/1997	52
08	68		Ovary	Papillary adenocarcinoma	05/1994	5
09	62		Uterus	Leiomyosarcoma	05/1998	88
10	60		Peritoneum	Primary peritoneal carcinoma	09/1999	52
11	50		Right tibia	Leiomyosarcoma	10/1997	68
12	60		Ovary	Papillary serous adenocarcinoma	03/1994	114
13	49		Lung	Adenocarcinoma	04/1998	40
14	45		Renal	Spindle cell carcinoma	12/1999	114
15	63				08/1999	114
16	73		Ovary	Papillary adenocarcinoma	11/1991	88
17	58				11/1998	68
18	58	PH1/092 (A5B7)	Colon	Adenocarcinoma	05/2003	45
19	60				02/1999	45
20	73				11/2001	45
21	57	PH1/066	Uterus	Leiomyosarcoma	07/1998	N/A
22	70	PH1/092 (A5B7)	Colon	G2 Adenocarcinoma	10/2003	45
23	66			Adenocarcinoma	08/2001	45
24	32				11/2002	45

**Table 2:** Angiogenic expression antibody profile

**Antibody**	**Type of marker**	**Source**	**Concentration**	**Specificity and non-specific staining**	**Control sample**
CD34	Vascular	Novocastra NCL-END	1 : 60	Endothelium, connective tissue, erythrocytes	Tonsil
αSMA		Sigma A2547	1 : 20000	Smooth muscle	Tonsil
CD105		Dako M3527	1 : 1000	Proliferating endothelium, activated macrophages	Human breast carcinoma
CD61		Novocastra NCL-CD61-308	1 : 100	Endothelium, platelets, megakaryocytes, monocytes, macrophages	Tonsil, human placenta
VEGF		Neomarkers MS-1467	1 : 100	121,165, 189 Isoforms	Human breast carcinoma
Ki-67	Proliferation	Dako M7240	1 : 200	345, 395 Isoforms. nuclear expression	Tonsil
Glut-1	Hypoxia	Dako A3536	1 : 200	Erythrocytes, tumour	Tonsil
PDGF	Vascular/tumour/stromal	Novocastra NCL-PDEGF P-GF.44c	1 : 60	Tumour, macrophages, stroma, glia, epithelia	Tonsil, human placenta
Angiopoietin -1		Santa Cruz Biotech Inc. H-98 (sc-8357)	1 : 50	Tumour, myocardium, vascular smooth muscle, lung, liver	Human placenta
Angiopoietin -2		Santa Cruz Biotech Inc. H-70 (sc-20718)	1 : 50	Tumour, ovary, placenta, uterus	Human placenta

SMAα=α-smooth muscle actin.

**Table 3:** Results–angiogenic expression profile of 24 human tumours

**Patient ID**	**CD34 MVD per mm^2^**	**SMA PCI (%)**	**CD105 MVD per mm^2^**	**VEGF**	**Ki-67 (%)**	**Glut-1 (%)**	**Ang-1**	**Ang-2**	**PDGF**	**CD61 MVD per mm^2^**
01	153.67	31.70	6.88	0	ND	0.5	ND	ND	ND	ND
02	96.33	66.85	20.64	1	0.5	1.0	3	1	0	47.02
03	182.34	54.58	64.22	0	20.0	0.5	1	2	0	18.35
04	97.48	85.73	53.90	2	15.0	15.0	2	3	2	19.50
05	127.29	73.69	57.34	2	30.0	10.0	1	2	0	26.38
06	26.38	47.50	13.76	0	1.0	1.0	3	3	0	11.47
07	157.11	50.99	11.47	1	10.0	1.0	1	1	0	19.50
08	92.89	24.67	5.73	1	15.0	20.0	1	1	0	9.17
09	137.61	59.03	10.32	0	10.0	1.0	2	1	0	17.20
10	96.33	41.54	38.99	2	50.0	30.0	0	2	1	43.58
11	151.38	72.51	59.63	0	2.0	0.5	1	2	0	10.32
12	60.78	55.58	19.50	2	40.0	30.0	0	1	0	6.88
13	83.72	57.14	10.32	2	5.0	0.5	1	1	0	1.15
14	160.55	20.53	76.83	2	10.0	5.0	1	2	0	29.82
15	150.23	30.60	30.96	1	15.0	25.0	1	3	0	19.50
16	110.09	77.43	27.52	0	15.0	1.0	2	1	2	11.47
17	123.85	57.90	29.82	2	20.0	5.0	2	3	0	5.73
18	65.37	66.37	24.08	3	60.0	20.0	1	3	0	16.06
19	128.44	44.58	16.06	2	30.0	10.0	1	2	0	24.08
20	90.60	80.15	26.38	2	30.0	5.0	2	2	0	6.88
21	79.13	32.63	36.70	3	1.0	60.0	1	3	1	27.52
22	82.57	79.83	11.47	2	40.0	25.0	2	2	0	9.17
23	142.09	60.85	3.40	3	40.0	2.0	1	1	0	8.52
24	178.90	53.00	73.39	2	25.0	15.0	3	3	1	12.61

MVD=microvessel density; ND=not done, due to lack of available tumour tissue; PCI=pericyte coverage index. For the categorical variables, 0=negative; 1=weak expression; 2=moderate expression; 3=strong expression.

**Table 4:** Percentage change in DCE-MRI kinetic parameters 4 h following infusion of CA4P

**Patient ID**	**CA4P Dose (mg m^−2^)**	**% Change *K*^trans^ post-CA4P**	**% Change IAUGC_60_ post-CA4P**
04	40	63.27	15.66
13		15.02	**186.00**
18	45	−22.91	−8.38
19		26.53	17.99
20		39.71	31.94
23		−30.32	−30.03
24		36.51	29.43
01	52	−29.56	−12.26
06		65.75	**146.30**
07		7.38	−23.81
10		**−67.14**	−58.92
02	68	−30.61	−19.23
05		1.59	−7.32
11		−0.35	−17.02
17		111.84	−15.44
03	88	**−75.20**	−57.65
09		−58.02	−58.26
16		33.01	−1.88
12	114	**−65.49**	**−90.21**
14		−20.00	−13.87
15		93.46	**82.38**

Statistically significant positive or negative values in bold. There is no obvious visual relationship between CA4P dose and fall in kinetic parameters, or correlation with vascular markers. There are three missing patients: 08 CA4P dose too low (5 mg m^−2^), 21 WF no data available, 22 EH severe motion artefact – data unusable.

**Table 5a:** Correlation coefficients (*r*^2^) and statistical significance between continuous variables and MRI parameters (*n*=21)

	**Continuous immunohistochemical variable**					
**MRI parameter**	**CD34 MVD**	***α*SMA PCI**	**CD105 MVD**	**CD61 MVD**	**Ki-67 (%)**	**Glut-1 (%)**
*K*^trans^ per min	*r*^2^<−0.001	*r*^2^=−0.079	*r*^2^<−0.001	*r*^2^=−0.011	*r*^2^=−0.077	*r*^2^=0.018
	*P*=0.98	*P*=0.23	*P*=0.957	*P*=0.539	*P*=0.233	*P*=0.488
IAUGC_60_ mM Gd per s	*r*^2^<−0.001	*r*^2^=−0.095	*r*^2^=−0.002	*r*^2^<−0.001	*r*^2^=−0.011	*r*^2^=0.036
	*P*=0.899	*P*=0.185	*P*=0.852	*P*=0.925	*P*=0.478	*P*=0.422

IAUGC=initial area under the gadolinium concentration-time curve; MVD=mean microvessel density; PCI=pericyte coverage index; SMA=smooth muscle actin.

The majority of calculations yield a negative regression line ie fall in MRI parameter following CA4P is greater if higher MVD.

**Table 5b:** ANOVA test for relationships between categorical variables and MRI parameters

	**Categorical variable**			
**MRI parameter**	**Ang-1**	**Ang-2**	**PDGF**	**VEGF**
*K*^trans^ per min	F=0.965	F=3.812	F=0.197	F=1.654
	d.f. (3,15)	d.f. (2,16)	d.f. (2,16)	d.f. (3,16)
	*P*=0.435	*P*=0.044	*P*=0.823	*P*=0.217
IAUGC_60_ mM Gd per s	F=1.284	F=1.728	F=0.468	F=2.064
	d.f. (3,15)	d.f. (2,16)	d.f. (2,16)	d.f. (3,16)
	*P*=0.316	*P*=0.209	*P*=0.635	*P*=0.145

F-value, degrees of freedom, and *P*-value are shown.
